# SERS-Based Methodology for the Quantification of Ultratrace Graphene Oxide in Water Samples

**DOI:** 10.1021/acs.est.2c00937

**Published:** 2022-06-14

**Authors:** Elena Briñas, Viviana Jehová González, María Antonia Herrero, Mohammed Zougagh, Ángel Ríos, Ester Vázquez

**Affiliations:** † Department of Organic Chemistry, Regional Institute of Applied Scientific Research (IRICA), 13071 Ciudad Real, Spain; ‡ Department of Organic Chemistry, Faculty of Science and Chemistry Technologies, University of Castilla-La Mancha (UCLM), 13071 Ciudad Real, Spain; § Department of Analytical Chemistry and Food Technology, Faculty of Pharmacy, University of Castilla-La Mancha (UCLM), 02071 Albacete, Spain; ∥ Department of Analytical Chemistry and Food Technology, University of Castilla-La Mancha (UCLM), 13071 Ciudad Real, Spain

**Keywords:** Raman spectroscopy, SERS, graphene oxide, quantification, water samples

## Abstract

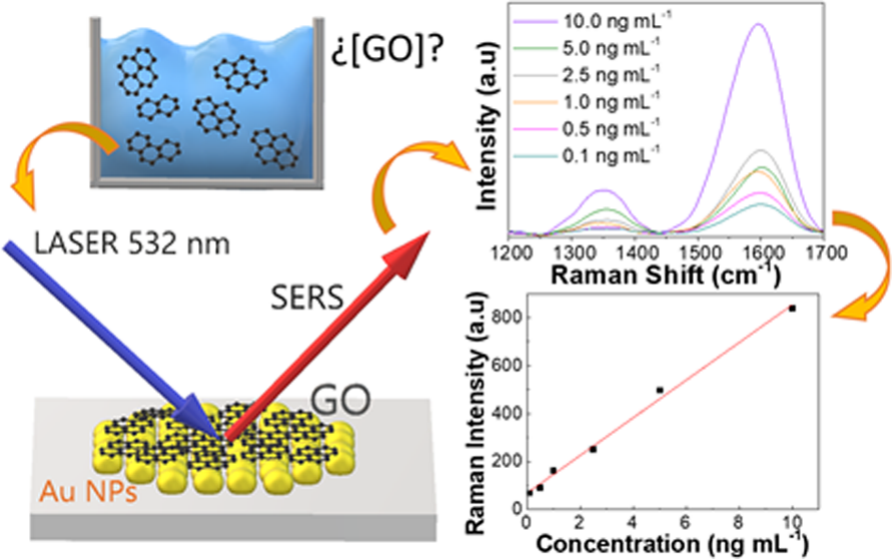

The extensive use of graphene materials in real-world applications has increased their potential release into the environment. To evaluate their possible health and ecological risks, there is a need for analytical methods that can quantify these materials at very low concentrations in environmental media such as water. In this work, a simple, reproducible, and sensitive method to detect graphene oxide (GO) in water samples using the surface-enhanced Raman spectroscopy (SERS) technique is presented. The Raman signal of graphene is enhanced when deposited on a substrate of gold nanoparticles (AuNPs), thus enabling its determination at low concentrations with no need for any preconcentration step. The practical limit of quantification achieved with the proposed method was 0.1 ng mL^–1^, which is lower than the predicted concentrations for graphene in effluent water reported to date. The optimized procedure has been successively applied to the determination of ultratraces of GO in water samples.

## Abstract 2

This study describes how to detect very small amounts of graphene-based materials in aquatic environments in relation to materials applications and policy regulations.

## Introduction

The discovery of graphene and graphene-related materials (GRM) has been a great boost for materials science. Their extraordinary characteristics, such as excellent mechanical and optical properties, and thermal and electrical conductivities,^1−4^ have led to them being used, either alone or in composite materials, in numerous applications.^5−7^ Indeed, these uses are so different that they range from energy generation and storage systems to sensors, nanocomposite fabrics, or even biological applications.^8^ This rapid development means that there are already many different GRM-based products on the market. For example, they have been used in epoxy resins to improve their mechanical properties, in conductive inks for flexible electronics, in anticorrosion coatings, as part of interconnectors for 5G data communication, sports products (bicycles, rackets, or shoes)^9^ or wearable health monitors.^10−12^

In parallel to the development of these applications, the techniques used for GRM characterization have also improved,^13^ but even today, one of the issues that remains to be resolved is their detection and quantification in low concentrations in environmental media while differentiating them from other carbonaceous materials. Throughout their life cycle, these graphene-based systems can end up in the environment, for example, in aqueous systems, and cause health problems and even become harmful to ecosystems due to absorption by organisms.^14,15^ Some health-related problems could be pulmonary inflammation or thrombotoxicity in contact with high concentrations of carbon nanomaterials.^16^ As such, the detection of graphene materials in these media is crucial for evaluating their potential ecotoxicological effects.^17,18^

Paradoxically, GRMs, and in particular graphene oxide (GO), can be used in environmental protocols for water treatment.^19^ GO has been widely used for the preparation of membranes, providing new opportunities for high-performance nanofiltration applications.^20^ This nanofiltration can be used for decolorization^21,22^ water softening,^23−25^ natural organic matter removal,^26^ heavy-metal removal,^27^ and organic solvent separation.^28,29^ Moreover, GO has shown bactericidal properties, and flakes of GO can envelop and confine microorganisms by enclosing them in an insulating carbon blanket.^30^ Despite all of these advantages, GO could be released from the membranes and contaminate the treated waters.

For all of these reasons, and to establish the safe use of graphene in everyday products, it is necessary to develop simple, rapid, and sensitive methods to analyze and detect GO in water samples at ultratrace levels. Analytical techniques for quantifying GO are normally based on its unique physicochemical properties, which differentiate GO from other compounds.^13^ These approaches take advantage of the structural, thermal, and electrical properties of GO and include X-ray photoelectron spectroscopy (XPS), UV–vis spectroscopy, fluorescence spectroscopy, and Raman spectroscopy. Although the XPS technique is widely used for characterizing and, at times, detecting GO in water samples, it is subject to interference from the abundant, naturally occurring carbon-containing substances in waters, and therefore is not very useful for quantifying GO in water. With regard to the UV–vis spectroscopy, GO can be quantified using the Lambert–Beer law;^31^ however, this technique cannot distinguish the absorbance of GO from that of other substances, thus leading to false positives. Similarly, fluorescence spectroscopy is not used as widely as UV–vis spectroscopy to quantify GO in aqueous media due to the nonlinear relationship between fluorescence intensity and the concentration of GO in aqueous media, thus meaning that this technique is mainly used for semiquantitative analysis. Finally, isotope labeling is not suitable for practical samples.^32^

Raman spectroscopy offers better specificity than XPS, UV–vis spectroscopy, and fluorimetry for identifying GO by detecting the signature defect (*D*, ∼1350 cm^–1^) and graphitic (*G*, ∼1580 cm^–1^) bands representative of the sp^2^-hybridized network of carbon disrupted by edges and defects along the basal plane. However, this technique is not sensitive enough to detect the amount of GO present in real samples. In this regard, Yang et al.^33^ developed a method for the fast identification and quantification of GO in local water samples based on reduction with hydrazine to minimize the fluorescence signal and subsequent analysis using Raman spectroscopy, obtaining a limit of quantification (LOQ) of 1000 ng mL^–1^. To improve the sensitivity, GO in water samples has been subjected to a preconcentration step on a cellulose membrane and finally determined by fluorescence quenching using graphene quantum dots,^34^ giving an LOQ of 117 ng mL^–1^. However, these methods are less sensitive and more time-consuming owing to the need for reduction and preconcentration steps, respectively. These shortcomings can be circumvented by developing effective and selective methods for minimizing the problems associated with sample treatment and sensitivity. Surface-enhanced Raman spectroscopy (SERS) is a good alternative for detecting GO with high selectivity and sensitivity. Indeed, given its potential for extremely high enhancement levels, SERS transforms Raman spectroscopy from a structural analytical tool to a structurally sensitive single-molecule probe.^35^ When GO is in contact with metallic nanoparticles, two effects may contribute to the enhancement of the Raman signal. First, the collective oscillation of electrons in metallic nanoparticles, like gold nanoparticles (AuNPs), leads to the so-called plasmon resonance, which can resonate with the energy of the excitation laser, thus resulting in high local optical fields (electromagnetic field enhancement). In addition, the electronic interaction between graphene and metal nanostructures can result in an increase in the Raman signal (chemical enhancement).^35−38^ AuNPs have advantages over other metal nanoparticles due to their easy synthesis and excellent stability, providing a high surface/volume ratio with exceptional optoelectronic properties. These properties can also be controlled by varying their size, shape, and the surrounding chemical environment, being crucial to achieve better SERS substrates.^39−41^

The enhancement factor (EF) is a helpful tool to estimate the efficiency of SERS substrate as an amplifier of the Raman signal of the analytes.^42^ This factor defines the intensity enhancement phenomenon observed in the Raman signal due to the presence of the appropriate SERS substrate.^43,44^ The SERS phenomenon can enhance inelastic light scattering events by a factor of 10^6^ or more, thus allowing researchers to develop sensitive methods for quantifying several molecules and compounds.^36^

Herein, we describe a new and simple method for detecting and quantifying GO in aqueous samples using SERS. This method has good reproducibility for aqueous samples and high sensitivity, thereby improving the reported limit of detection (LOD) for GO by fluorescence sensors.^34^ The results are very promising as regards the development and commercialization of GO-based devices.

## Experimental Section

### Materials and Methods

All reagents were of analytical grade or better. Graphene oxide (p50–100 mesh, p/n 1800) was supplied by Abalonyx (Oslo, Norway). Sodium citrate (≥99%), gold(III) chloride (≥99.99%), 2-propanol (≥99.8%), and hydrochloric acid (≥37%) were obtained from Sigma-Aldrich (St. Louis, MO). Nitric acid (≥60%) was purchased from Panreac (Barcelona, Spain). Acetone (≥99.5%) was acquired from Labkem (Barcelona, Spain). Si/SiO_2_ wafers were purchased from Pure Wafer (San José, California). Graphene oxide working solutions were prepared at room temperature by dispersion in deionized water.

Raman spectra were recorded using an InVia Renishaw microspectrometer equipped with a 532 nm point-based laser. The power density was kept below 10%, and a 1 s acquisition time was used to avoid laser heating effects. The spectrum range was between 1109.18 and 2228.67 cm^–1^. The resulting spectrum was obtained by averaging 3000 spectra in mapping mode. Scanning electron microscopy (SEM) images were recorded using a ZEISS GeminiSEM 500 field emission instrument (Zeiss, Germany) with an acceleration voltage of 0.02–30 kV and a probe current of 3 pA to 20 nA. This instrument is equipped with several detectors: an in-lens secondary electron detector, an in-lens energy selected backscatter detector (EsB), an annular scanning transmission electron microscopy (STEM) detector (aSTEM 4), and an EBSD detector (electron backscatter diffraction) to investigate crystalline orientation. The samples were prepared on a lacey carbon surface by deposition of a dispersion of the nanomaterial. The ultraviolet–visible (UV–vis) spectrum of gold nanoparticles was collected using 1 cm quartz cuvettes and a Cary 5000 UV–vis–NIR spectrophotometer in the range of 200–800 nm at room temperature. An ultrasound bath (Selecta, Barcelona, Spain), an Ossila spin coater (United Kingdom), and a Milli-Q system (Millipore, Bedford, MA) were also used. Thermogravimetric analysis (TGA) was carried out with a TGA Q50 (TA Instruments) equipment using a ramp from 100 to 800 °C at 10 °C min^–1^ under a nitrogen atmosphere. LECO CHNS-932 analyzer was used for the elemental analysis with complete combustion of the sample.

#### Synthesis of Gold Nanoparticles

All glass materials used were cleaned using aqua regia (a mixture of 1:3 nitric acid and hydrochloric acid), then washed with ultrapure water, and dried in air. The nanoparticles were synthesized following a previously described procedure.^45^ Briefly, 5 mL of a 1 mM solution of gold(III) chloride was heated to boiling under magnetic stirring. Then, 5 mL of a 38.8 M sodium citrate solution was added at the boiling point and the mixture was heated at reflux for 15 min. Upon stirring, the solution became wine-red in color. Finally, the colloidal suspension was stirred and then allowed to stand at room temperature, giving a final concentration of 10.7 nM in water. AuNPs were kept in an ambered bottle at 4 °C.

#### Characterization of Gold Nanoparticles and Graphene Oxide

**Figure 1:**
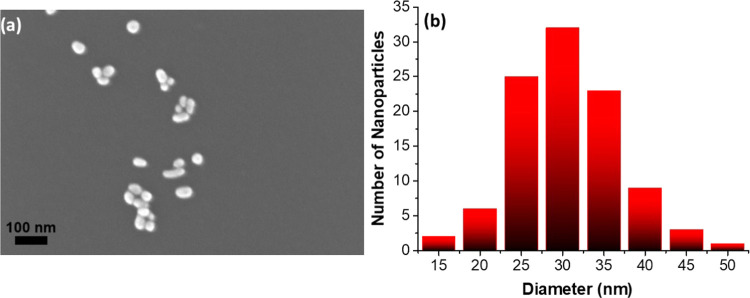
(a) STEM images and (b) size diameter distributions (100 measurements) of synthesized AuNPs.

The gold nanoparticles synthesized were characterized by UV–vis spectroscopy and STEM to determine their size and concentration (Figure 1). The STEM image shows very homogeneous spherical nanoparticles with an average diameter of around 27.31 ± 6.5 nm. The concentration of the stock solution (10.7 nM) was calculated from the UV–vis band at 520 nm using the Lambert–Beer law.^46^

**Figure 2:**
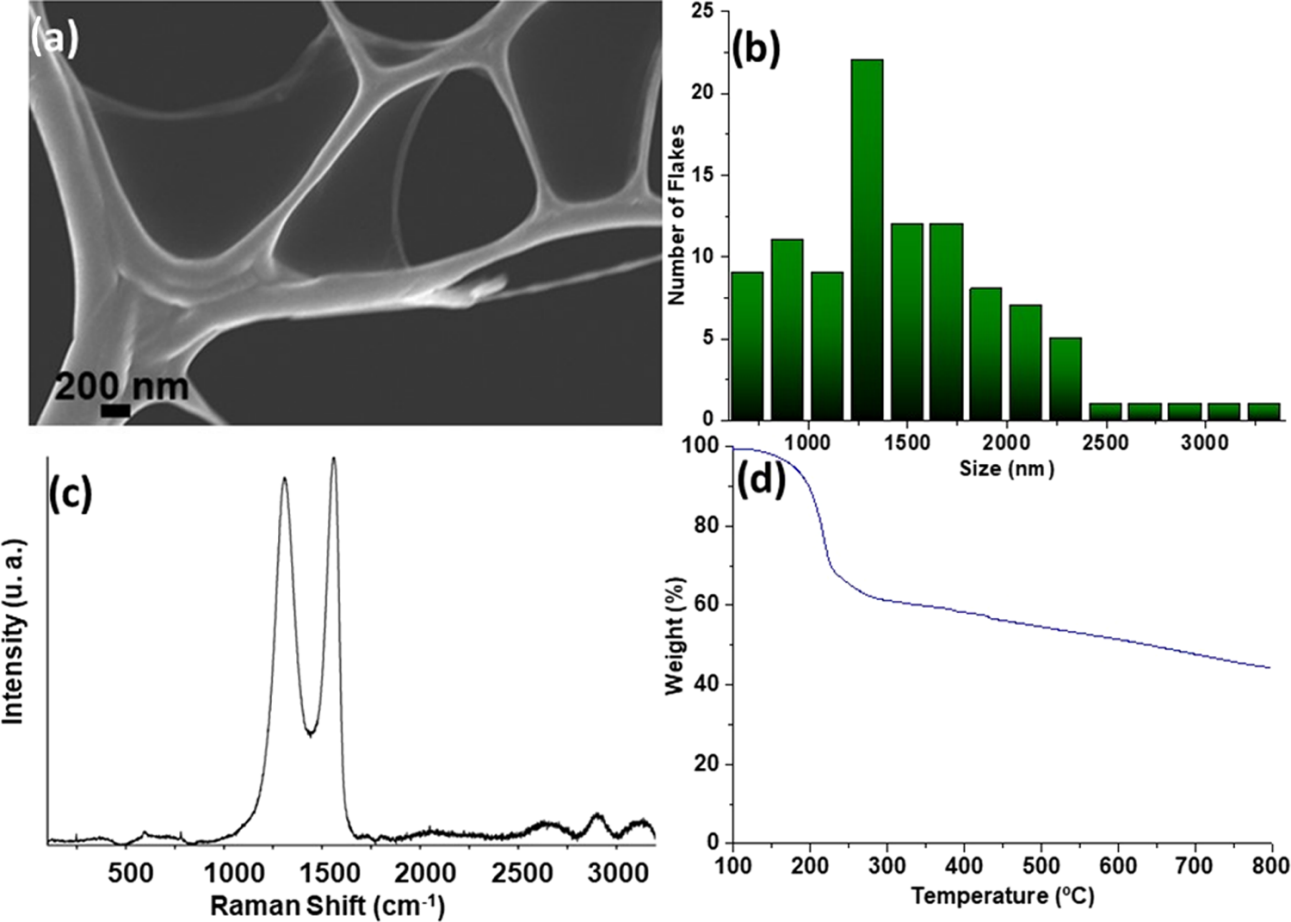
(a) STEM image, (b) size distributions of layers, (c) Raman spectrum of GO, and (d) thermogravimetric analysis of GO.

Commercial GO was characterized by recording the STEM and Raman spectra. The STEM image (Figure 2a,b) shows thin layers with an average size of 1339.96 ± 557.14 nm. The characteristic Raman spectrum of GO exhibits the different characteristic bands (Figure 2c). Thus, the D peak, which is located around 1346.08 cm^–1^, is related to the defect and disorder level within the layers, whereas the G peak, at 1580 cm^–1^, is due to structures with sp^2^ domains.^47,48^ Regarding the GO oxidation degree, a weight loss of 48.6% was obtained by thermogravimetric analysis (Figure 2d). Elemental analysis gave average values of C (45.52 wt %), H (2.60 wt %), N (0.08 wt %), S (1.47 wt %), and O (50.33 wt %), in agreement with the loss observed by TGA.

#### SERS Measurements

The SERS-active substrate was prepared as follows: 20 μL of a 10.7 nM solution of the previously synthesized AuNPs was deposited dropwise (two drops of 10 μL each) onto a Si/SiO_2_ substrate previously cleaned with piranha water (1:3 H_2_O_2_:H_2_SO_4_), 2-propanol, and acetone, and dried under a nitrogen gas flow. Afterward, 120 drops (1200 μL) of GO solution were added to the center of the SERS substrate using an automatic micropipette. These drops were deposited individually, waiting until complete evaporation of the previous drop, heating at 50 °C using a hot plate.

The analyte deposited was analyzed by SERS detection using a Raman spectrometer with a 532 nm point-based laser. The laser power was set to 50 mW, and an acquisition time of 1 s was employed for measurements. Three different substrates were prepared for each GO concentration. The intensity of the GO band at 1346.08 cm^–1^ was selected as the analytical signal and subsequently used to quantify GO in the water samples. The spectrum of AuNPs deposited on the substrate by drop casting (20 μL of a 10.7 nM solution) (Figure S1) was used as the control and was subtracted for all samples to avoid interference of the Au signal with the GO band. All measurements and the calibration line were performed on the same day and 24 h after the preparation of the substrates. SERS measurements were acquired for 400 μm^2^ surfaces located in the middle of the drops with an average of 3000 spectra measurements. The baseline was removed using Windows-based Raman Environment (WiRE) software. The 3000 spectra were then averaged to give a single spectrum for each replica using a program generated in MATLAB R2020a with our own code. Finally, calculations were performed using OriginPro 9.1.

## Results and Discussion

### Optimization of the Preparation of the SERS Substrate and Measurements

To design a robust and reliable methodology for the quantification of GO, different variables were studied. The first step involved selection of the appropriate substrate for deposition of the AuNPs. Si/SiO_2_ was chosen since it shows a strong peak at 520 cm^–1^ in the Raman spectrum. This band is helpful for calibration due to the lack of interference with GO.^46^ The optimal parameters for the Raman studies were a laser power of 50 mW and a wavelength of 532 nm. The sensitivity of the spectrum and duration of the acquisition was 1 s, the same conditions for graphene-related materials as previously established by our group.^49^ Although the best results were obtained by mapping 10,000 points, we reduced the spectra to 3000 points to optimize the time per measurement. The mapped areas have an average surface of 400 μm^2^.

The next step was to find the optimal volume of AuNPs to obtain a homogeneous SERS substrate (to improve the repeatability and reproducibility of our methodology) and, therefore, the best improvement in the GO signal. Our group has already optimized a volume of AuNPs of 20 μL (10.7 nM) for carbon nanomaterials, and we have shown that a higher concentration of AuNPs decreases the Raman signal of graphene due to the thickness of the gold layer.^46^

**Figure 3:**
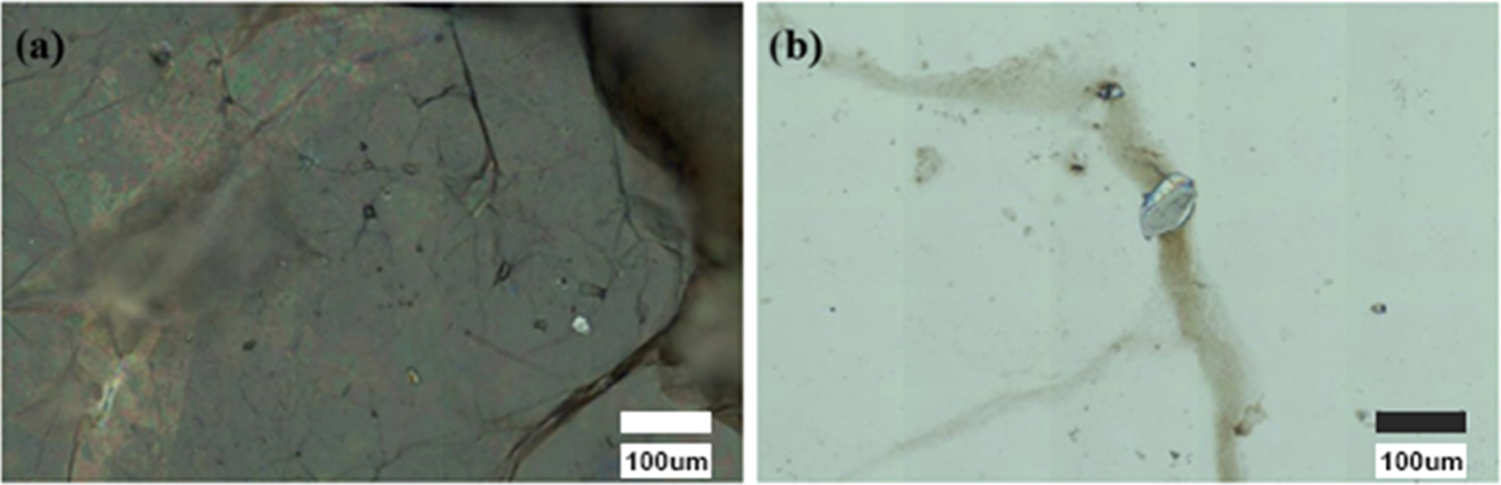
Images of SERS substrates taken with a Raman microscope: 20 μL (2 drops of 10 μL) of AuNP solutions (10.7 nM) and 600 μL of GO dispersions (50,000 ng mL^–1^) deposited by: (a) drop casting and (b) spin coating.

The best methodology for the deposition of both AuNPs and GO to achieve a homogeneous surface was also studied. Two procedures, namely, drop casting and spin coating, were examined. Each drop was deposited and evaporated for the first method, heating at 50 °C on a hot plate. The procedure was repeated until all of the desired drops had been placed. For spin coating, the drops were deposited, and the substrate spun at 3000 rpm for 20 s between drops using a spin coater. The highest homogeneity was obtained using drop casting with the same number of drops (Figure 3).

Two substrates were prepared using drop casting: one by adding 20 μL of AuNPs and 600 μL of GO dispersion (2000 ng mL^–1^) and another by adding 600 μL of GO dispersion (2000 ng mL^–1^) only. We found an increase in the intensity of the GO peaks in the Raman spectra in the presence of Au nanoparticles (Figure S2).

**Figure 4:**
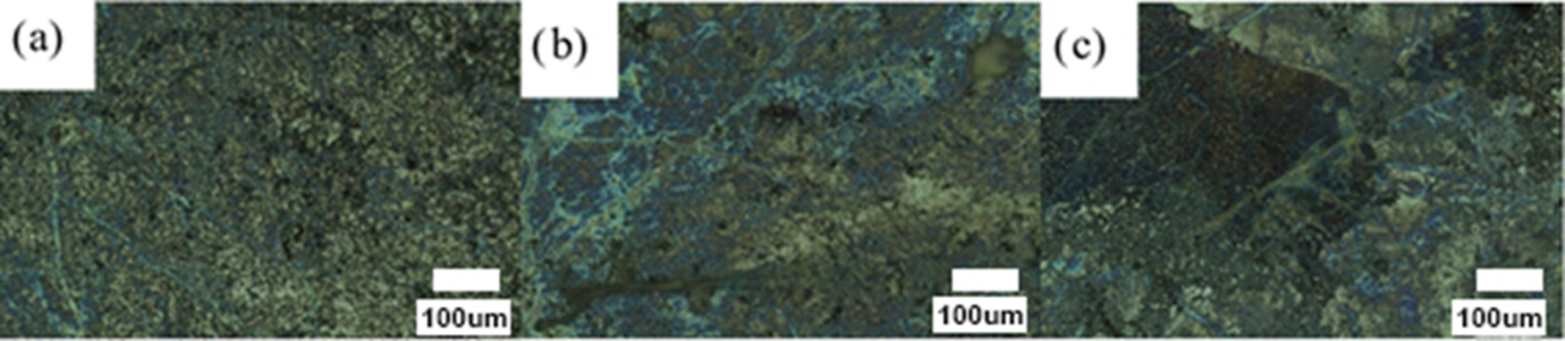
Images of SERS substrates taken with the Raman microscope: 1200 μL of GO dispersion was deposited at different concentrations: (a) 30 ng mL^–1^; (b) 20 ng mL^–1^; (c) 1 ng mL^–1^.

Subsequently, different volumes (300, 600, and 1200 μL) of GO standards (30, 20, and 1 ng mL^–1^) were deposited on the substrate using the drop casting technique to determine the optimal volume of GO needed for detection. The best result in terms of homogeneity was at 120 drops (1200 μL). Even when obtaining homogeneous surfaces (Figure 4), the best correlation between concentration and intensity was found in the middle of the surface, with the repeatability between different substrates increasing.

Finally, the optimal band for the quantification of GO was also examined. The D peak (1346.08 cm^–1^) shows a good correlation between intensity and concentration; on the contrary, the G peak (1587.22 cm^–1^) does not show correlation. The SERS substrate presents a highly variable peak intensity at the same Raman shift as for the G peak of GO, with a relative standard deviation (RSD) of 6.7%. However, for the band at 1346.08 cm^–1^ (D peak), the control AuNP substrate shows an RSD of 2.6%, with a very low intensity. As such, the D band was chosen as the most appropriate peak to achieve repeatable and reproducible measurements.

### Raman Enhancement of the SERS Spectra of Graphene Oxide

An increase in the D and G bands for GO can be observed in Figure S2, which shows the SERS spectra of GO obtained when AuNPs are deposited on its substrate compared to the Raman spectra of GO on a Si/SiO_2_ plate with no AuNPs. The enhancement factor for the hybrid substrate can be calculated using the following equation^50^
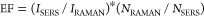
where the intensity *I* is the height of the GO band at 1346.08 cm^–1^ and *N* represents the total number of analyte molecules deposited on the substrate. Instead of the number of molecules, we have used the mass of GO deposited on the substrate.

SERS enhancement factor of the AuNP substrate was obtained with the comparison of the Raman signal for 1200 μL of a 10 ng mL^–1^ solution of GO deposited onto a bare Si/SiO_2_ substrate and 1200 μL of a 0.1 ng mL^–1^ solution of GO onto the AuNP substrate. Figure S3 shows that the D band of graphene appears at 1346.08 cm^–1^ and the G band appears at 1587.22 cm^–1^.

An enhancement factor of 61 was determined for our aqueous graphene dispersions. The EF value is not standardized, and extremely different EF factors can be observed depending on the substrate and the analyte. Examples in the literature were not found for the quantification of GO by the SERS technique in real water samples, but the quantification of some presynthesized graphene derivatives is described. For example, the SERS spectra of single-layer graphene, prepared by mechanical exfoliation (“Scotch-tape method”^51^), were measured by deposing Au nanoparticles on a graphene substrate by thermal evaporation.^52^ In another recent study, an enhancement of the Raman signals for as-grown graphene on Cu foils was observed by depositing a 4 nm thick Au film.^53^ Similar orders of magnitude were found in the quantification of these presynthesized graphene derivatives using more sophisticated methods.^52,53^

### Analytical Features of the Proposed Method

**Figure 5:**
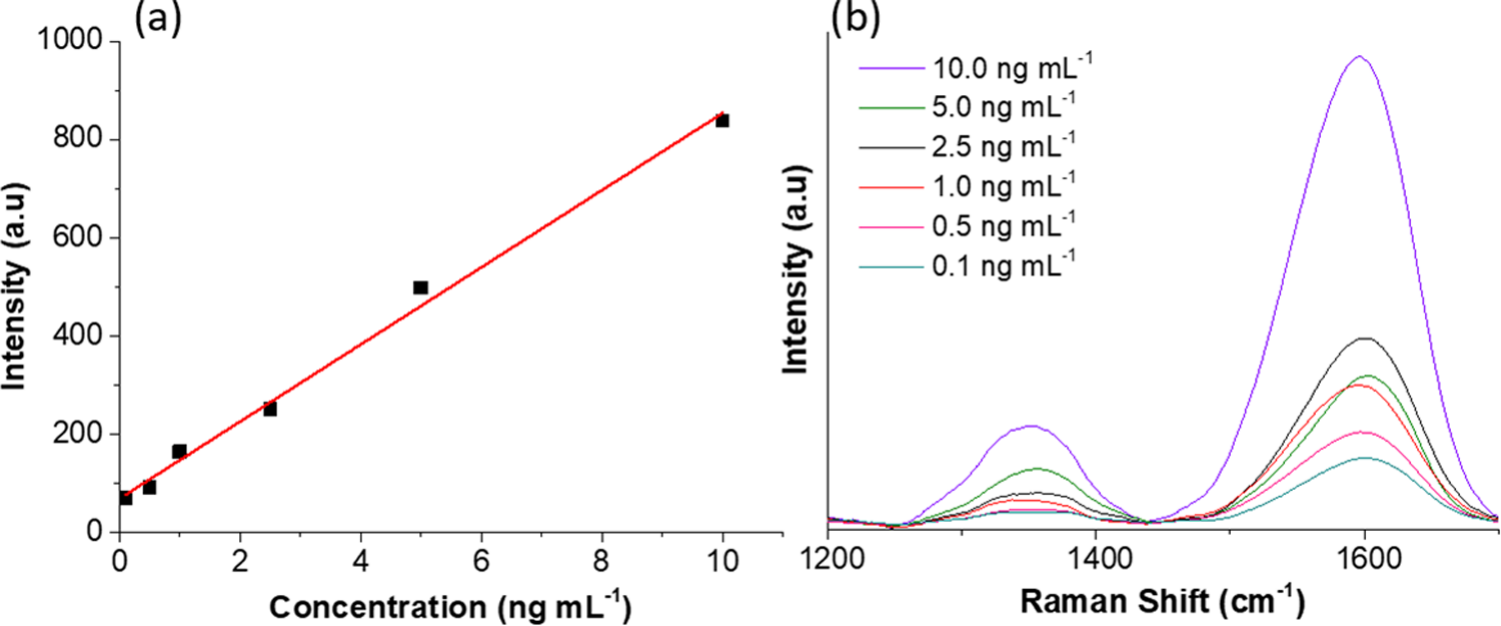
(a) Calibration curve and (b) SERS spectra at different GO concentrations in the range of 0.1–10 ng mL^–1^.

**Figure 6:**
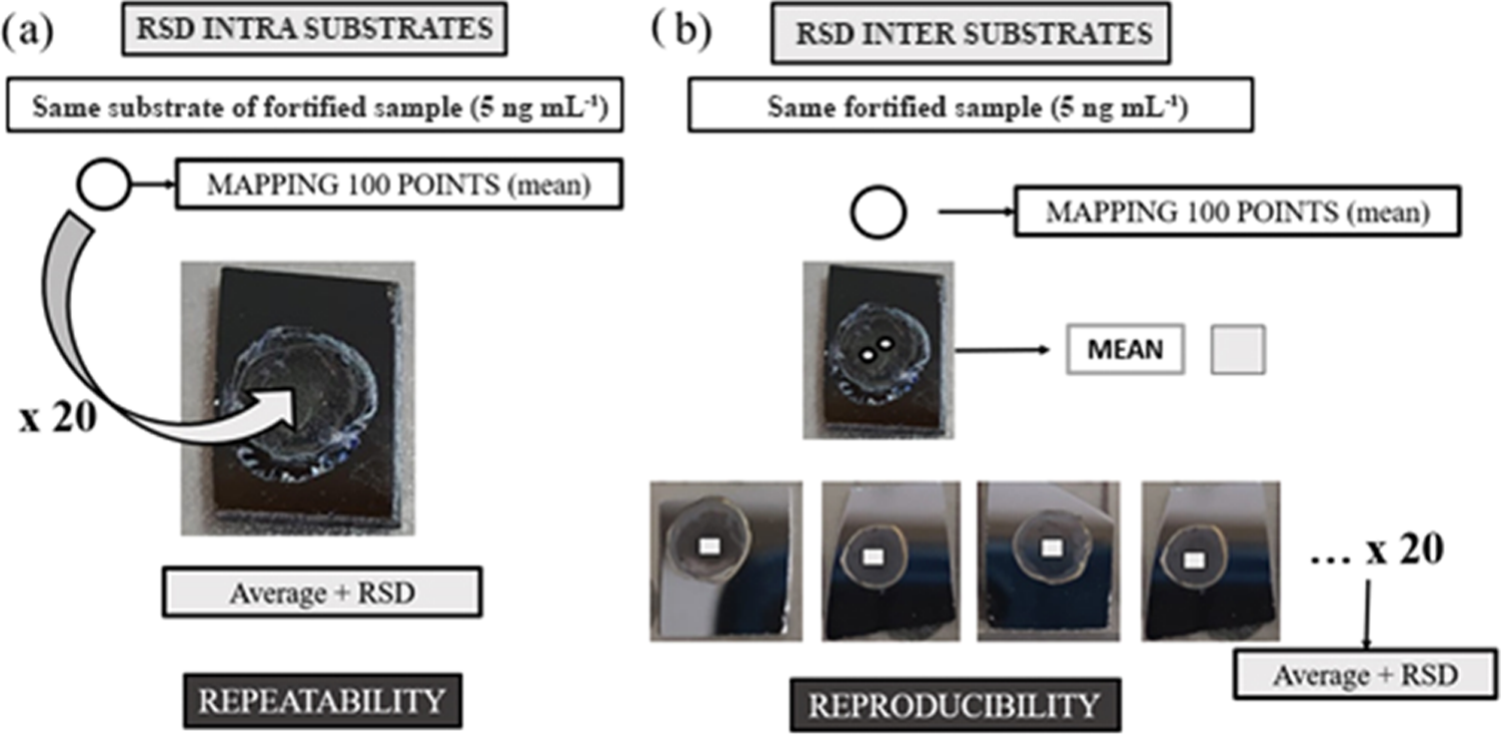
Scheme for the calculation of (a) repeatability, relative standard deviation between measurements on the same substrate (RSD_intra_substrate); (b) reproducibility, relative standard deviation between measurements on different substrates at the same concentration (RSD_inter_substrate).

The analytical performance of the proposed method was studied to evaluate its utility for quantitative analysis. The SERS-active substrate was evaluated in terms of sensitivity [limit of detection (LOD) and practical limit of quantification (P-LOQ)] and precision. The analytical features were calculated by depositing the optimal volume of AuNPs (20 μL) on a bare Si/SiO_2_ plate, using a volume of 1200 μL for all concentrations tested. The analytical parameters are summarized in Table 1. An external calibration curve using the analytical signal (intensity) peak at 1346.08 cm^–1^ was constructed for the concentration range of 0.1–10 ng mL^–1^ (Figure 5). A straight line with an *R*^2^ value of 0.995 was obtained, thus demonstrating that the proposed method can be used for quantitative analytical purposes. The LODs, defined as the concentration of analyte giving a signal equivalent to the control signal plus 3 times its standard deviation (SD), are also presented in Table 1. The intercept value and its corresponding SD for the calibration equation were used to calculate this value. The LOD value obtained was 0.11 ng mL^–1^. Repeatability and reproducibility tests were also carried out. These aspects were estimated by calculating the RSDs between the measurements for the same substrate and measurements for different substrates at the same concentration. Thus, we differentiated between intrasubstrate RSD and intersubstrate RSD (Figure 6). The intrasubstrate RSD was determined from the average value of 20 measurements of the same SERS substrate with a 5 ng mL^–1^ GO solution deposited on it. Each of these 20 measurements is the result of averaging 100 spectral measurements (Figure 6a). The intersubstrate RSD (Figure 6b) was determined from the average value of two measurements of 20 different SERS substrates on which the same 5 ng mL^–1^ GO solution was deposited, that is, 20 replicates of the same solution at the same concentration. Each of these two measurements per substrate results from averaging 100 spectra from 100 different locations on the same substrate. As such, the intrasubstrate RSD indicates the repeatability and the intersubstrate RSD indicates the reproducibility. The intrasubstrate RSD was 5.60%, and the intersubstrate RSD was 8.65%. Our method therefore shows promising results in terms of repeatability and reproducibility, with values of less than 10% when using the Raman technique.

**Table 1:** Analytical Features of the Method for Determining GO in Water

parameter	value
linear range (ng mL^–1^)	0–10.00
*y* = (*a* ± *S*_a_)*X* + (*b* ± *S*_b_)	*y* = (78.537 ± 13.554)*X* – (68.483 ± 2.884)
*R*^2^	0.995
LOD^a^ (ng mL^–1^)	0.11
P-LOQ^b^ (ng mL^–1^)	0.10
RSD intrasubstrate^c^ (%)	5.60 (*n* = 20)
RSD intersubstrate^d^ (%)	8.65 (*n* = 20)

a Limit of detection.

b Practical limit of quantification.

c Relative standard deviation, determined from 20 measurements at a GO concentration of 5 ng mL^–1^ for the same substrate.

d Relative standard deviation, determined from the average value of 20 measurements at a GO concentration of 5 ng mL^–1^ using 20 different substrates.

The P-LOQ, which is defined as the minimum level at which GO can be determined in water samples with acceptable accuracy (>80%) and precision (RSD < 10%), was 0.1 ng mL^–1^ (Table 1). This value is much lower than the reported LOQ of 10^3^ and 116.69 ng mL^–1^ using Raman spectroscopy and fluorescence spectroscopy, respectively, with previous preconcentration steps.^33,34^

### Application to the Determination of GO in Water Samples

To validate the proposed method, deionized and tap water samples were analyzed following the recommended procedure. Although no graphene oxide was expected to be found in them, the recoveries obtained were higher than those anticipated for spiked samples. Three fortification levels were assayed for each sample; the recovery values are shown in Table 2. The recoveries obtained ranged from 95.66 to 105.28%, depending on the concentration level and type of sample. Three replicate samples were measured at each concentration to evaluate the accuracy of the method.

**Table 2:** Recovery of GO from Deionized and Tap Water Samples

sample	added (ng mL^–1^)	found (ng mL^–1^)	recovery (%)	RSD (%)
deionized water	0.25	0.25	100.43	3.22
	3.50	3.68	105.28	2.72
	7.00	6.87	98.09	3.94
tap water	0.25	0.25	100.47	7.19
	3.50	3.35	95.66	3.88
	7.00	6.93	99.13	6.49

The RSD was calculated from the average recoveries calculated for the three replicates of each sample. Thus, the RSD range for recovery values was from 2.72 to 7.19%.

The continuous increase in the number of products based on graphene derivatives makes it likely that, at the end of their useful life, these materials will end up in the environment. However, due to the insufficient information regarding toxicological aspects, their regulation is still scarce. For this reason, in this study, we developed a method to detect and quantify GO using SERS. This analytical technique allows us to detect analytes down to the single-molecule level, with high specificity, thus resulting in a methodology that is able to quantify GO at a trace level. We have also shown that the presence of AuNPs increases the Raman spectra signal of GO, thus allowing the detection of this compound without a previous preconcentration step.

The method proposed has a limit of quantification of 0.1 ng mL^–1^. Our literature review failed to identify methodologies with this sensitivity and power to detect low concentrations. Moreover, this study has shown how this methodology is simple, useful, and effective in water samples. Although the preparation of substrate and sample are carried out off-line, we are already considering the on-line preparation together with the optimization of new active SERS substrate. Subsequent progress in the development of methodologies for detecting and quantifying GO rapidly and straightforwardly in the environment will help to regulate these products and ensure their safe use in everyday products.

## Supporting Information Available

Raman spectra of the control, AuNPs (20 μL of a 10.7 nM solution) (Figure S1), Raman spectra of GO (2000 ng mL^–1^) on a Si/SiO_2_ surface (black line) and on a substrate prepared with 20 μL of a 10.7 nM solution of AuNPs (red line) (Figure S2), and Raman spectra of GO (10 ng mL^–1^) on a Si/SiO_2_ surface (black line) and GO (0.1 ng mL^–1^) on a substrate prepared with 20 μL of a 10.7 nM solution of AuNPs (red line) (Figure S3) (PDF)
