# Supplementary material for: SERS-Based Methodology for the Quantification of Ultratrace Graphene Oxide in Water Samples
Source: Environ Sci Technol. 2022 Jun 14;56(13):9527–35. doi: 10.1021/acs.est.2c00937 (PMC9261266; doi:10.1021/acs.est.2c00937)
Supplement: Supplementary file 1 — es2c00937_si_001.pdf [file es2c00937_si_001.pdf]

Supporting Information for

"A SERS-based methodology for the quantification of ultra-trace graphene oxide in water samples"

Elena Briñas,[‡a] *Viviana Jehová González,*[‡a] *María Antonia Herrero,*[a, b,*] *Mohammed Zougagh,*[a, c] *Ángel Ríos,*[c,d] *Ester Vázquez.*[a, b,*]

a. Department of Organic Chemistry, Regional Institute of Applied Scientific Research (IRICA), 13071 Ciudad Real, Spain

b. Department of Organic Chemistry, Faculty of Science and Chemistry Technologies, University of Castilla-La Mancha (UCLM), 13071 Ciudad Real, Spain

c. Department of Analytical Chemistry and Food Technology, Faculty of Pharmacy, University of Castilla-La Mancha (UCLM), 02071 Albacete, Spain

d. Department of Analytical Chemistry and Food Technology, University of Castilla-La Mancha (UCLM), 13071 Ciudad Real, Spain

‡These authors contributed equally to this work

*Corresponding author.

e-mail: mariaantonia.herrero@uclm.es; Ester.Vázquez@uclm.es

This supporting information contains 5 pages, 3 figures.

Figure S1. Raman spectra of the control, AuNPs (20 µL of a 10.7 nM solution)……..S3

Figure S2. Raman spectra of GO (2000 ng mL) on an Si/SiO_2 surface (black line) and on a substrate prepared with 20 µL of a 10.7 nM solution of AuNPs (red line)……………..S4

Figure S3. Raman spectra of GO (10 ng mL^{-1}) on a Si/SiO_2 surface (black line) and GO (0.1 ng mL-1) on a substrate prepared with 20 µL of a 10.7 nM solution of AuNPs (red line)…………………………………………………………………………………..S5

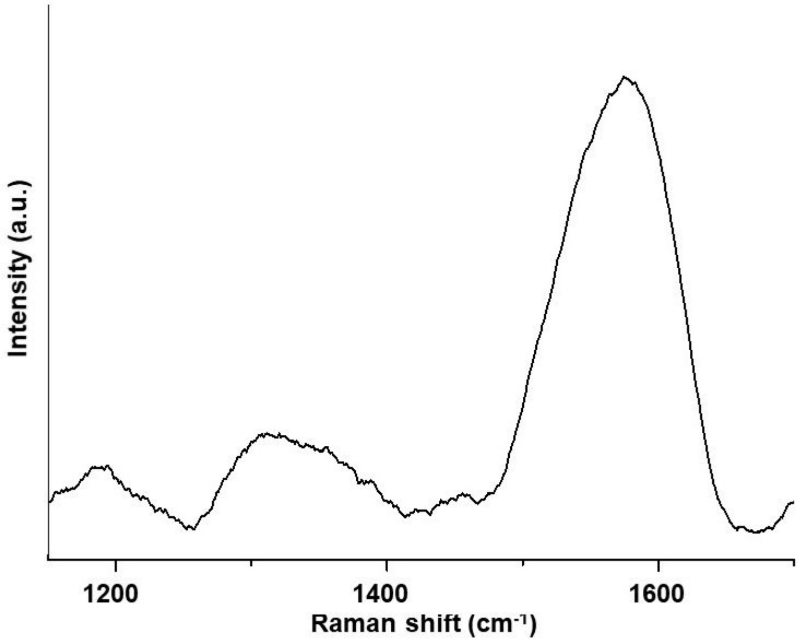

Figure S1. Raman spectra of the control, AuNPs (20 μL of a 10.7 nM solution).

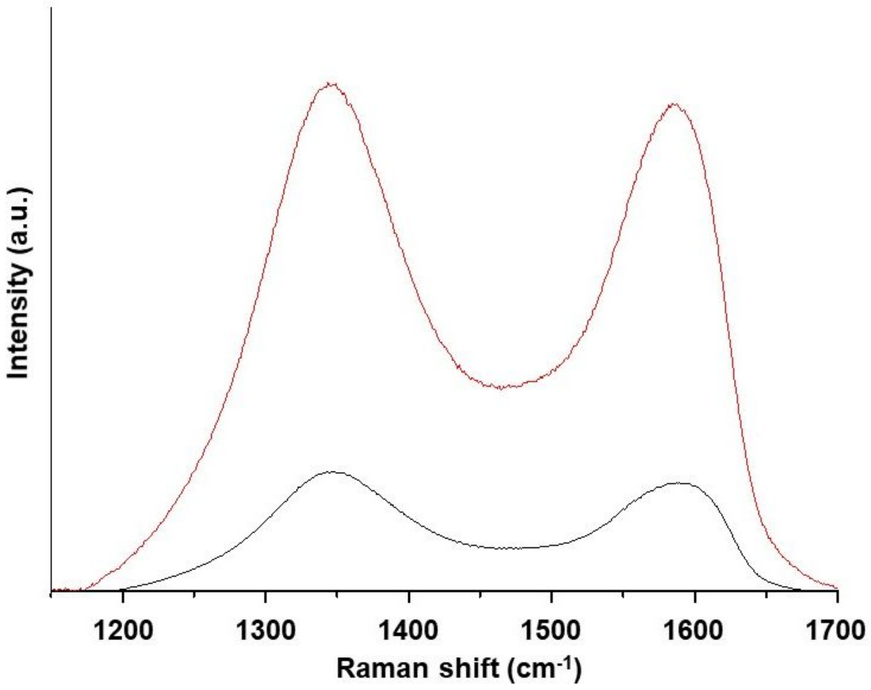

Figure S2. Raman spectra of GO (2000 ng mL) on an Si/SiO_2 surface (black line) and on a substrate prepared with 20 µL of a 10.7 nM solution of AuNPs (red line).

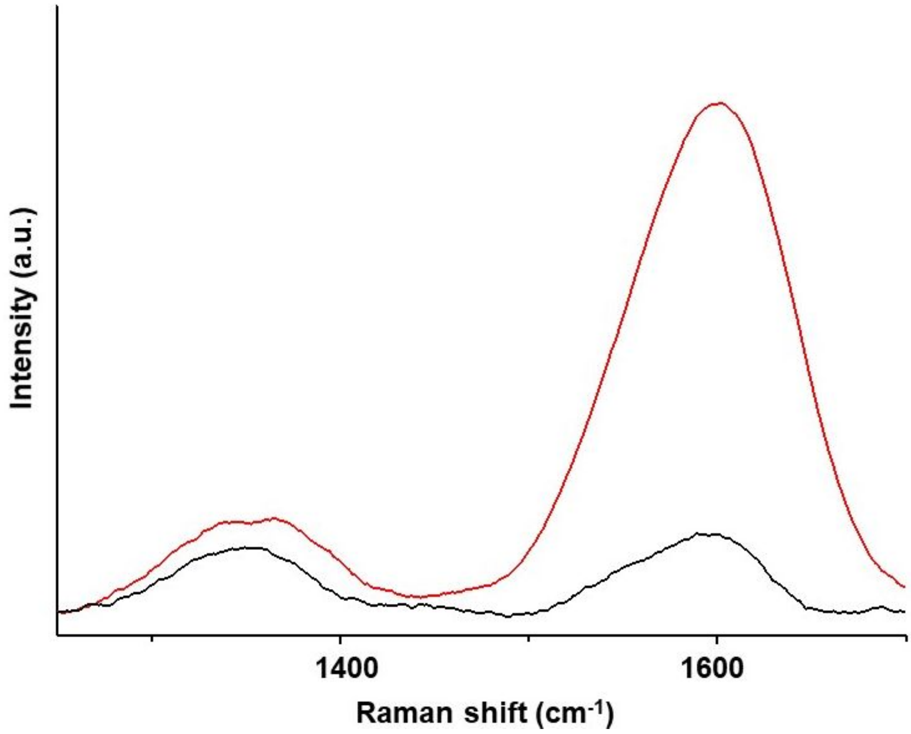

Figure S3. Raman spectra of GO (10 ng mL^{-1}) on a Si/SiO_2 surface (black line) and GO (0.1 ng mL^{-1}) on a substrate prepared with 20 μL of a 10.7 nM solution of AuNPs (red line).
